# Development and evaluation of a lyophilization protocol for colorimetric RT-LAMP diagnostic assay for COVID-19

**DOI:** 10.1038/s41598-024-61163-7

**Published:** 2024-05-09

**Authors:** Nayra Oliveira Prado, Anelis Maria Marin, Larissa Araujo Lalli, Heloisa Bruna Soligo Sanchuki, Denise Kusma Wosniaki, Jeanine Marie Nardin, Hugo Manoel Paz Morales, Lucas Blanes, Dalila Luciola Zanette, Mateus Nóbrega Aoki

**Affiliations:** 1 Laboratory for Applied Science and Technology in Health, Carlos Chagas Institute, Oswaldo Cruz Foundation (Fiocruz), Prof. Algacyr Munhoz Mader 3775 Street, Curitiba, 81350-010 Brazil; 2 Erasto Gaertner Hospital, Dr. Ovande Do Amaral 201 Street, Curitiba, Paraná 81520-060 Brazil

**Keywords:** Lyophilization, RT-LAMP, Colorimetric kit, COVID-19, Biochemistry, Biological techniques, Molecular biology

## Abstract

Molecular diagnostics involving nucleic acids (DNA and RNA) are regarded as extremely functional tools. During the 2020 global health crisis, efforts intensified to optimize the production and delivery of molecular diagnostic kits for detecting SARS-CoV-2. During this period, RT-LAMP emerged as a significant focus. However, the thermolability of the reagents used in this technique necessitates special low-temperature infrastructure for transport, storage, and conservation. These requirements limit distribution capacity and necessitate cost-increasing adaptations. Consequently, this report details the development of a lyophilization protocol for reagents in a colorimetric RT-LAMP diagnostic kit to detect SARS-CoV-2, facilitating room-temperature transport and storage. We conducted tests to identify the ideal excipients that maintain the molecular integrity of the reagents and ensure their stability during room-temperature storage and transport. The optimal condition identified involved adding 5% PEG 8000 and 75 mM trehalose to the RT-LAMP reaction, which enabled stability at room temperature for up to 28 days and yielded an analytical and diagnostic sensitivity and specificity of 83.33% and 90%, respectively, for detecting SARS-CoV-2. This study presents the results of a lyophilized colorimetric RT-LAMP COVID-19 detection assay with diagnostic sensitivity and specificity comparable to RT-qPCR, particularly in samples with high viral load.

## Introduction

In 2020, the novel coronavirus, known as SARS-CoV-2 (severe acute respiratory syndrome coronavirus 2), the causative agent of COVID-19 (coronavirus disease 2019), precipitated one of the most severe global health crises in recent history, mobilizing the scientific and medical communities to develop effective treatment and diagnostic protocols^1^. Prior to the initiation of vaccination campaigns, testing for the diagnosis and isolation of infected individuals was the primary strategy to prevent the disease's spread. Currently, testing tools are essential for various reasons, including facilitating international travel. Among these tools, nucleic acid amplification tests (NAT) and rapid antigen tests are employed, with the former being the more sensitive method and preferred for diagnostic confirmation^2^.

NAT for SARS-CoV-2 identification utilizes polymerase chain reaction (PCR) with reverse transcription by real-time PCR as the gold standard methodology^3^. However, these tests require robust equipment, trained personnel, and extensive time to complete the procedure^4^ In a health crisis scenario, the loop-mediated isothermal amplification technique with reverse transcription (RT-LAMP) emerged as an alternative for research and practical application, distinguished by its speed and practicality, especially for large-scale testing in resource-limited settings^2^. The RT-LAMP methodology can also be performed in a colorimetric version, allowing visual analysis and thus facilitating faster and more suitable point-of-care use^5^. However, transporting and distributing such tests to areas with limited infrastructure remains a challenge, as it involves logistics for transport and storage that require a cold chain due to the temperature sensitivity of most diagnostic components and their short shelf life^6^. The lyophilization process, a preservation method used in amplification reactions to make the mixtures resistant to environmental conditions, addresses this issue. While this procedure is common in commercial PCR kits, there are few strategies and evaluations for colorimetric RT-LAMP^7^. The advantages of the lyophilized product include storage at room temperature, increased product durability, ease of transportation (since it does not require temperature control), and conservation of material characteristics^7^.

An optimized lyophilization protocol requires extensive and complex screening of various reagents at different concentrations and formulations, alongside numerous other variables such as equipment and the time and temperature of freezing prior to lyophilization. This manuscript introduces a novel approach by utilizing several lyoprotectants, different lyophilization properties, and performance evaluation in SARS-CoV-2 clinical samples. It presents the results of the development, optimization, and validation of the RT-LAMP reaction lyophilization process for detecting SARS-CoV-2. This process includes a set of simple, accessible, and suitable additives for the protection of the reagents involved, maintaining the performance of a colorimetric RT-LAMP reaction, and increasing the shelf life of the finished product by ensuring the stability of the material even at room temperature.

## Results

### Lyophilization formulations evaluation

As part of the initial protocol screening for the lyophilization of the colorimetric SARS-CoV-2 RT-LAMP reaction, five protective reagents were tested in various combinations. For each substance, analyses focused on the effects of both the concentration and the absence of protective reagents in the reactions. Lyophilized samples with different compositions were compared to each other and to standard fresh reactions, which means SARS-CoV-2 RT-LAMP without any lyophilization process and submitted to 60 °C for 30 min along with lyophilized samples, containing 10^6^ copies of SARS-CoV-2 RNA and SARS-CoV-2 negative RNA control. This initial protocol was conducted using a standard lyophilization process, as described in the Methods section. In this phase, the results analyzed were visual, observing the final reaction color of the RT-LAMP, where yellow and red correspond to positive and negative samples, respectively.

The initial substance analyzed was trehalose, tested at four concentrations ranging from 50 to 150 mM in 10^6^ copies of SARS-CoV-2 RNA, SARS-CoV-2 negative RNA, and non-template control (NTC). We observed that trehalose at 75 mM yielded the best result. The second substance, arginine, was tested at eight concentrations ranging from 2.5 mM to 75 mM, with 10 mM showing the best performance. Both selected conditions displayed final reaction colors very similar to those of the fresh control reactions for both SARS-CoV-2 positive and negative templates. Following this initial observation, a combination of trehalose and arginine at 75 mM and 10 mM, respectively, was tested; however, the results indicated no significant difference compared to the isolated protective reagents.

Following the experimental design, Polyethylene Glycol (PEG) 2,000 and PEG 8,000 were lyophilized in different concentrations in combination with 75 mM trehalose and 10 mM arginine. Initially, PEG 2,000 was tested at four concentrations (ranging from 1 to 10%), with the 10% concentration demonstrating the best result when combined with both arginine and trehalose, compared to fresh controls. For PEG 8,000, the same tests were conducted, and the 5% concentration was identified as the most suitable. Subsequently, we prepared a combination of 10% PEG 2,000 and 5% PEG 8,000 in fresh control reactions for both SARS-CoV-2 positive and negative samples and observed no impact on amplification when assessing the final reaction color, indicating that PEG does not interfere with RT-LAMP. The last tested substance was Polyvinylpyrrolidone (PVP) 40,000, tested at 2% and 5% final concentrations in conjunction with 75 mM trehalose and 10 mM arginine. We found that all concentrations displayed inconsistent performance, leading to the decision to discard this polymer at this time. Table 1 summarizes the four compositions followed in the experiments.

**Table 1:** Four compositions of lyophilization for colorimetric SARS-CoV-2 RT-LAMP using arginine, trehalose, PEG 2000, and PEG 8000.

Condition	Arginine	Trehalose	PEG 2,000	PEG 8,000
1	10 mM	–	10%	–
2	10 mM	–	–	5%
3	–	75 mM	10%	–
4	–	75 mM	–	5%

**Figure 1:**
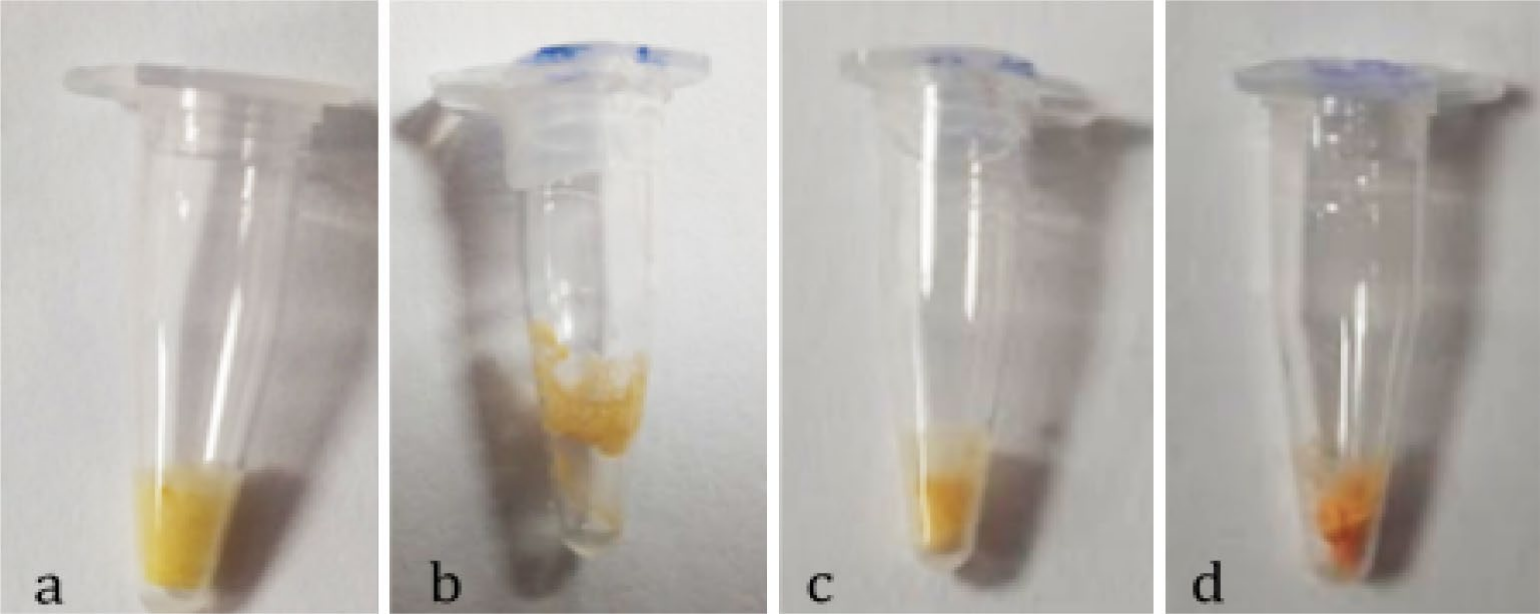
Representative image of the “cake” from colorimetric SARS-CoV-2 RT-LAMP lyophilized conditions as follows: (**a)**. 10 mM arginine + 10% PEG 2000; (**b)**. 75 mM trehalose + 10% PEG 2000; (**c)**. 10 mM arginine + 5% PEG 8000; (**d)**. 75 mM trehalose + 5% PEG 8000.

Another phase of lyophilization standardization and optimization involved a visual analysis of the appearance of the "cake," the term used to describe the lyophilized product. The inclusion of PEG and PVP aimed to enhance the cake's appearance, making it resemble a piece of dough adhering to the bottom of the container without spreading. As previously mentioned, we discarded the PVP 40,000 condition at this stage due to its repeated inconsistencies. Figure 1 below displays lyophilized samples with 10% PEG 2,000 and 5% PEG 8,000 combined with 75 mM trehalose and 10 mM arginine. Following this initial screening of lyophilization reagents, we focused on four compositions that exhibited similar performance to fresh controls for both SARS-CoV-2 positive and negative templates, indicating that these formulations effectively protected the Bst enzyme and colorimetric RT-LAMP compounds.

### Lyophilization time and color measurement

**Figure 2:**
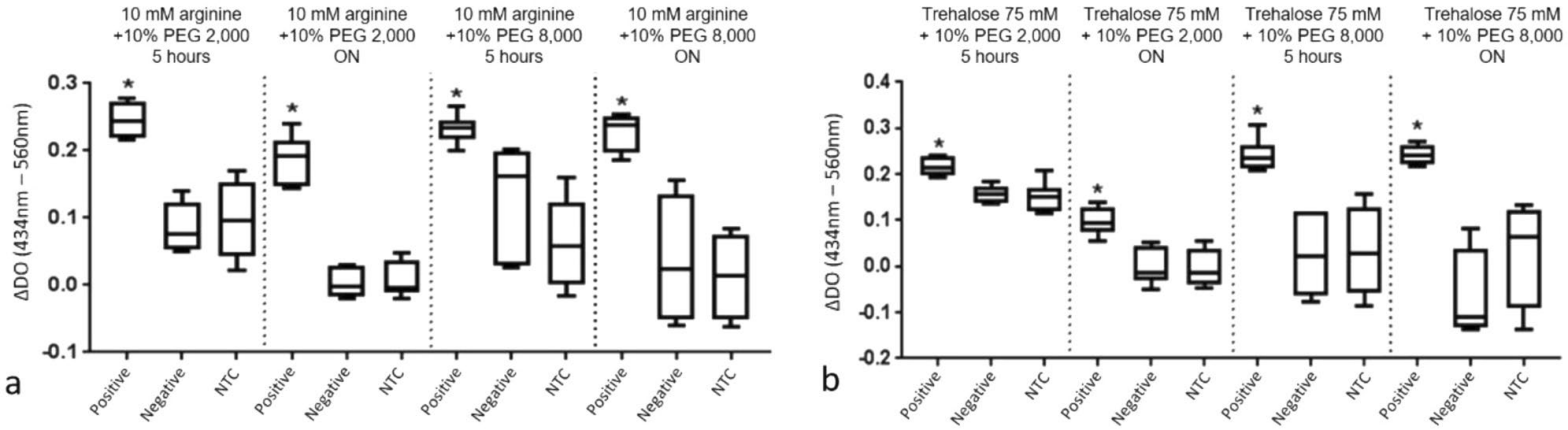
A and B—Result of spectrophotometry for four conditions and two lyophilization times, indicating the difference between absorbance by spectrophotometry at wavelengths of 434 nm and 560 nm (ΔDO), respectively, for positive SARS-CoV-2 samples, negative RNA and NTC. *p < 0.01, with A referring to conditions containing arginine and B referring to conditions containing trehalose.

After selecting four lyophilization conditions, the protocol focused on optimizing lyophilization time by comparing durations of five hours and overnight (1five hours and 30 min) to determine the best lyophilization time for the selected formulations. In each selected formulation from Table 1, the analysis involved, after lyophilization and resuspension, adding 10^6^ copies of SARS-CoV-2 RNA, SARS-CoV-2 negative RNA, and NTC. The same templates were added to fresh controls. Each group was lyophilized together and at the selected time. The protocol was repeated three times for each condition and duration, aiming to analyze the consistency of the results. After amplification, the samples were analyzed in a spectrophotometer at 434 and 560 nm to obtain quantitative data complementary to the visual analyses. We observed a statistically significant difference in the ΔOD (optical density) of positive samples for SARS-CoV-2 compared to NTC in all conditions (Fig. 2 and supplementary Fig. 1), while no difference was observed between samples using SARS-CoV-2 negative RNA and NTC. These results indicated three outcomes: first, a numerical difference in color between positive and negative SARS-CoV-2 RNA samples, minimizing naked-eye bias and demonstrating that the lyophilization process does not interfere with the colorimetric RT-LAMP reaction; second, the final reaction colors’ contrast and consistency were maintained according to reagent composition and templates; and third, the influence of time in the proposed lyophilization protocol was assessed.

In the tests conducted at this stage, we were able to observe and select conditions to follow when conducting the experiments. Parameters considered included greater color contrast between SARS-CoV-2 positive and negative samples (both negative RNA and NTC) and the reaction consistency, as shown by the amplitude and standard deviation results. Lyophilization time was analyzed using the same parameters. The conditions selected to continue the experiments were 10 mM arginine + 10% PEG 2000 and 75 mM trehalose + 5% PEG 8,000. Lyophilization time protocols showed no significant difference in colorimetric RT-LAMP performance. However, overnight lyophilization presented more problems and inconsistencies; therefore, the five-hour lyophilization period was selected for evaluation in subsequent steps.

### Limit of detection

**Figure 3:**
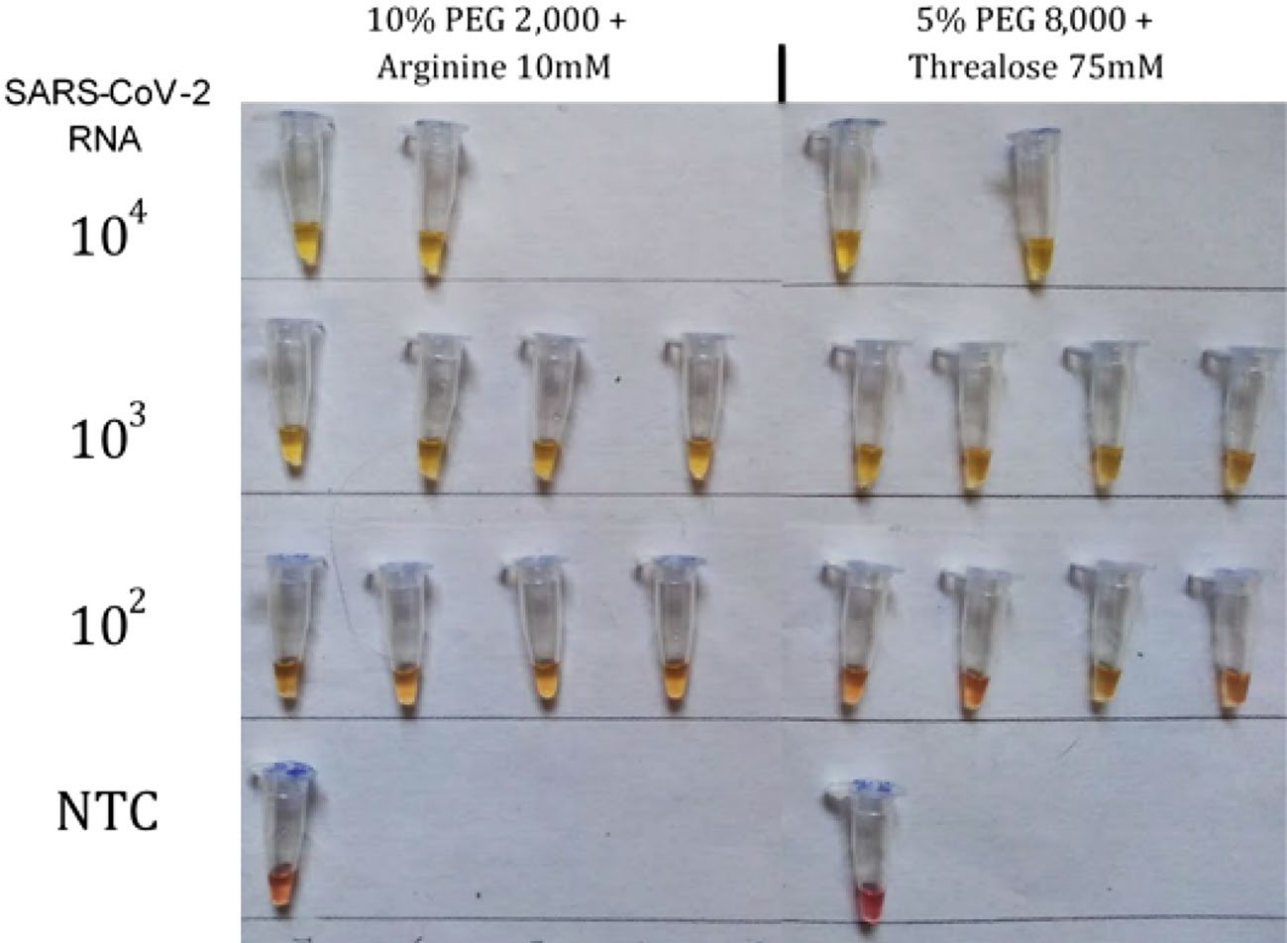
Representative image of 10% PEG 2,000 + 10 mM arginine and 5% PEG 8,000 + 75 mM trehalose colorimetric SARS-CoV-2 RT-LAMP lyophilized conditions with 10^4^, 10^3^, and 10^2^ SARS-CoV-2 RNA copies, demonstrating that with the first formulation, the color change occurs in the samples containing 10^2^ copies, while in the second formulation, amplification occurs only up to 10^3^ copies. *NTC* Non-template control.

At this stage, we optimized two lyophilization formulations over five hours to evaluate diagnostic performance. The primary parameter assessed was the approximate detection limit after lyophilization using the two formulations across four concentrations of SARS-CoV-2 RNA. We conducted three independent trials with 10^5^, 10^4^, 10^3^, and 10^2^ copies of SARS-CoV-2 RNA, each lasting five hours. The results indicated that all reactions containing 10^3^ copies of SARS-CoV-2 RNA tested positive under both lyophilization formulations. Specifically, the 75 mM trehalose + 5% PEG 8,000 condition resulted in a slightly orange-yellowish hue, indicative of a positive result yet clearly distinct from a negative outcome. Conversely, in the arginine-based formulation, amplification was observed up to 10^2^ copies of SARS-CoV-2 RNA in 50% of the reactions, with negative reactions exhibiting an intermediate orange coloration. In comparison, all reactions at the same RNA concentration using the trehalose 75 mM + 5% PEG 8,000 formulation tested negative (Fig. 3). Consequently, the approximate limit of detection (LoD) appears to be lower in the condition with 10 mM arginine + 10% PEG 2,000, ranging between 10^3^ and 10^2^ copies of SARS-CoV-2 RNA.

### Diagnostic performance

Following the colorimetric RT-LAMP lyophilized evaluation, the diagnostic performance was assessed using the two selected formulations in a five-hour lyophilization protocol. This approach allowed for the visualization of lyophilization performance on actual clinical samples characterized for SARS-CoV-2 presence by RT-qPCR. Fifty clinical samples from human swabs were utilized, of which 30 were SARS-CoV-2 positive and 20 were negative. Positive samples were subdivided into two groups based on the E-gene cycle threshold value (Ct): less than 29 and between 29 and 35. Samples with a Ct value less than 29 indicate a high viral load, while those between 29 and 35 represent a low viral load. Additionally, 20 samples undetected for the E-gene and with internal control amplification were tested.

**Figure 4:**
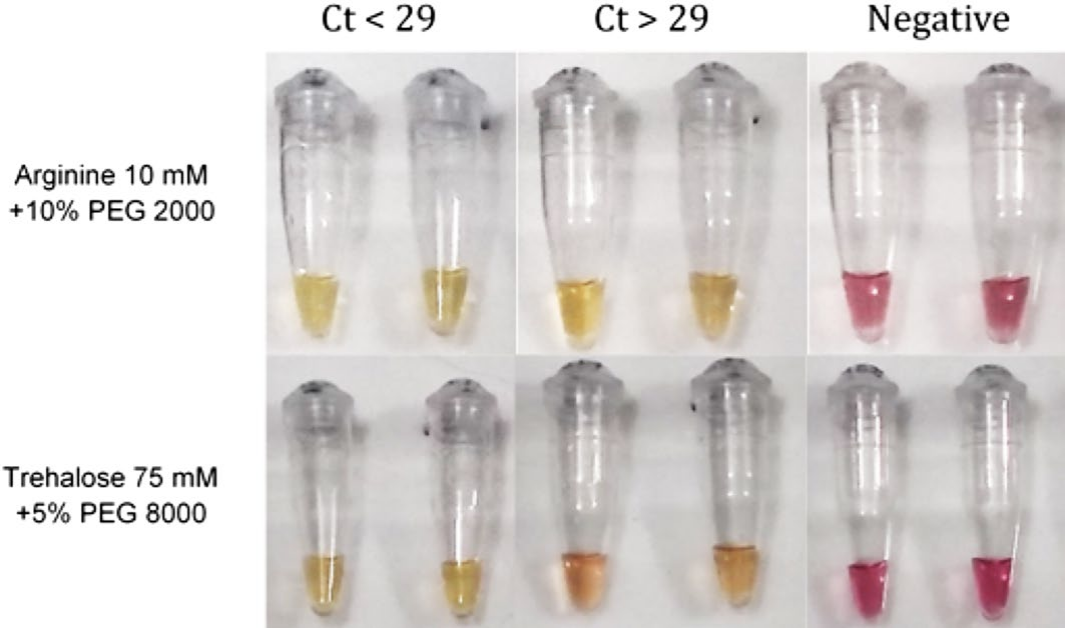
Representative image of 10% PEG 2000 + 10 mM arginine and 5% PEG 8000 + 75 mM trehalose colorimetric SARS-CoV-2 RT-LAMP lyophilized conditions with SARS-CoV-2 clinical samples, characterized by RT-qPCR, and subdivided into high (Ct < 29) and low (Ct > 29) viremia, and negative samples.

**Figure 5:**
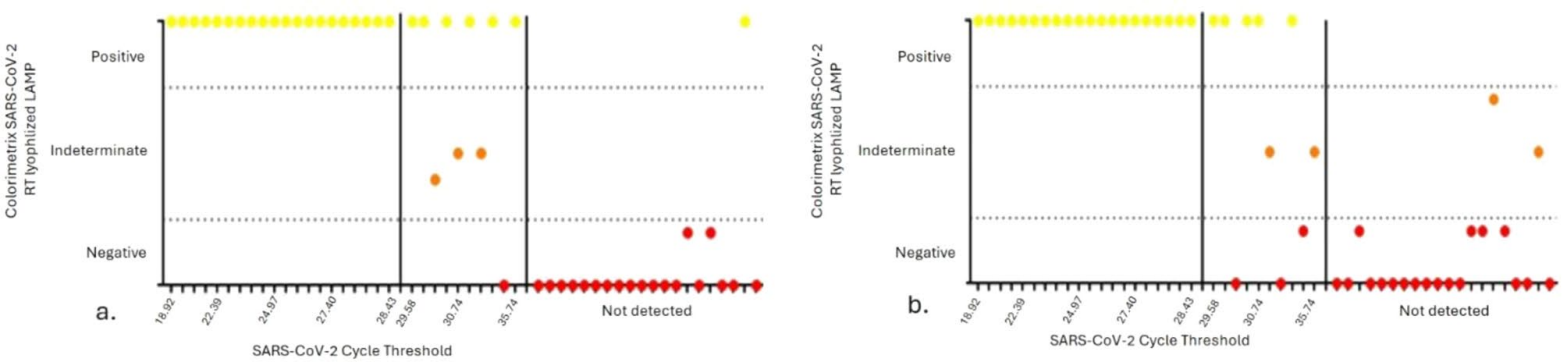
Schematic representation of the correlation between SARS-CoV-2 clinical sample detection by RT-qPCR and colorimetric SARS-CoV-2 RT-LAMP lyophilized: a. 10% PEG 2,000 + 10 mM arginine; b. 5% PEG 8,000 + 75 mM trehalose. Positive: samples exhibiting yellow coloration; intermediary: samples exhibiting orange coloration; negative: samples exhibiting red coloration.

The results are summarized in Table 2 and Figs. 4 and 5. We observed that all 20 clinical samples with Ct values lower than 29 for SARS-CoV-2, as detected by RT-qPCR in the condition with 10 mM arginine + 10% PEG 2,000, demonstrated amplification with corresponding color change. In the 75 mM trehalose + 5% PEG 8,000 condition, 19 samples amplified, and one sample turned reddish-orange, thus less defined but still clearly positive. For the 10 SARS-CoV-2 positive samples with a Ct value greater than 29, in the 10 mM arginine + 10% PEG 2,000 condition, six samples demonstrated amplification with corresponding color change, and three displayed an orange color. They were therefore indeterminate, and one remained red and was considered negative. In the 75 mM trehalose + 5% PEG 8,000 condition, five samples demonstrated amplification with corresponding color change; two displayed an orange color and were indeterminate, and three remained red, i.e., negative. These data demonstrate that when considering SARS-CoV-2 positive samples by standard RT-qPCR with a Ct value lower than 29, the diagnostic sensitivity was 100% for both lyophilization conditions. When considering positive SARS-CoV-2 samples, regardless of the Ct value, the diagnostic sensitivity was 86.6% and 83.33% for the 10 mM arginine + 10% PEG 2000 and 75 mM trehalose + 5% PEG 8000 conditions, respectively.

**Table 2:** Diagnostic sensitivity and specificity of Arginine 10 mM + 10% PEG 2000 and Trehalose 75 mM + 5% PEG 8000.

Samples	Arginine + 10% PEG 2000	Trehalose + 5% PEG 8000
	Total of samples/positive lyophilized RT-LAMP	Total of samples/positive lyophilized RT-LAMP
SARS-CoV-2 Ct < 29	20/20	20/20
SARS-CoV-2 Ct > 29	10/6	10/5
SARS CoV-2 negative	20/1	20/2

When analyzing SARS-CoV-2 negative samples using the 10 mM arginine + 10% PEG 2,000 and 75 mM trehalose + 5% PEG 8,000 protocols, we observed 19 and 18 yellow reactions, respectively, indicating negative results. This corresponds to a diagnostic specificity of 95% for the 10 mM arginine + 10% PEG 2,000 protocol and 90% for the 75 mM trehalose + 5% PEG 8,000 protocol. Notably, the false-positive samples in the 75 mM trehalose + 5% PEG 8,000 protocol displayed an orange color, indicating an indeterminate and not clearly positive result.

### Lyophilized colorimetric RT-LAMP stability

**Figure 6:**
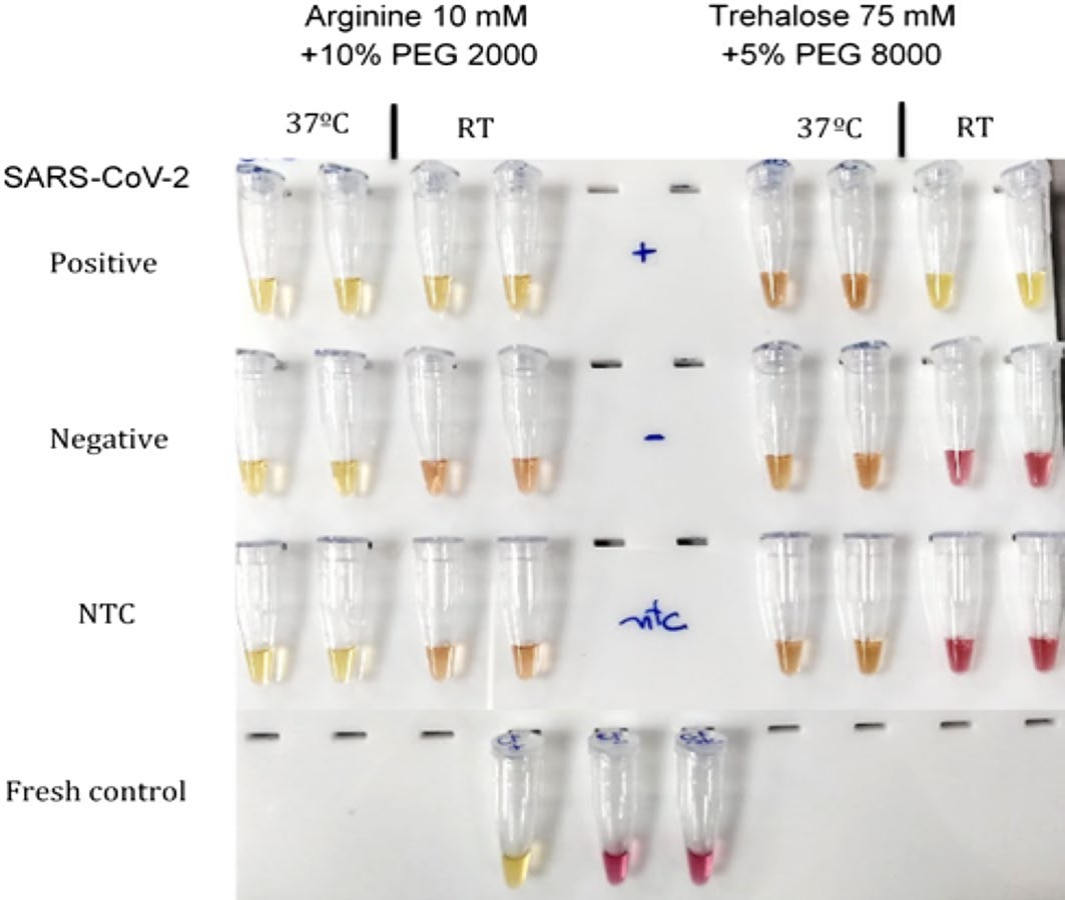
Week 1 of colorimetric SARS-CoV-2 RT-LAMP lyophilized conditions 10% PEG 2000 + 10 mM arginine and 5% PEG 8,000 + 75 mM trehalose. *RT* Room temperature, *NTC* Non-template control.

After evaluating the diagnostic performance, we assessed the stability of lyophilized colorimetric RT-LAMP, both at room temperature (20 °C) and 37 °C, using the 10 mM arginine + 10% PEG 2,000 and 75 mM trehalose + 5% PEG 8,000 formulations. At week 0, the lyophilized colorimetric RT-LAMP was resuspended and immediately tested with SARS-CoV-2 positive and negative RNAs following lyophilization. The appearance and results were consistent with previous tests, where the reaction was lyophilized and then resuspended and tested shortly after that. As described in the methods, seven subsequent tests were prepared for both room temperature and 37 °C with SARS-CoV-2 positive and negative RNAs. At the second time point (week 1), i.e., seven days after the start of the stability experiment, the lyophilized colorimetric RT-LAMP was resuspended, and SARS-CoV-2 positive and negative RNAs were added immediately before submitting to standard amplification. After resuspension, we noted that reactions with 10 mM arginine + 10% PEG 2,000 maintained at 37 °C showed a yellowish color, while those with 75 mM trehalose + 5% PEG 8,000 displayed a reddish color. Additionally, after 30 min in the thermocycler, both formulations showed no color change, indicating the loss of function of the reaction. Therefore, the two tested formulations of lyophilized colorimetric RT-LAMP demonstrated very low stability at 37 °C. For reactions kept at room temperature, both conditions initially showed a red color after resuspension and turned yellow after a standard thermal reaction with SARS-CoV-2 positive RNA, indicating a functional and stable reaction. However, the formulation with 75 mM trehalose + 5% PEG 8000 displayed better results with more contrast between positive and negative samples (Fig. 6).

**Figure 7:**
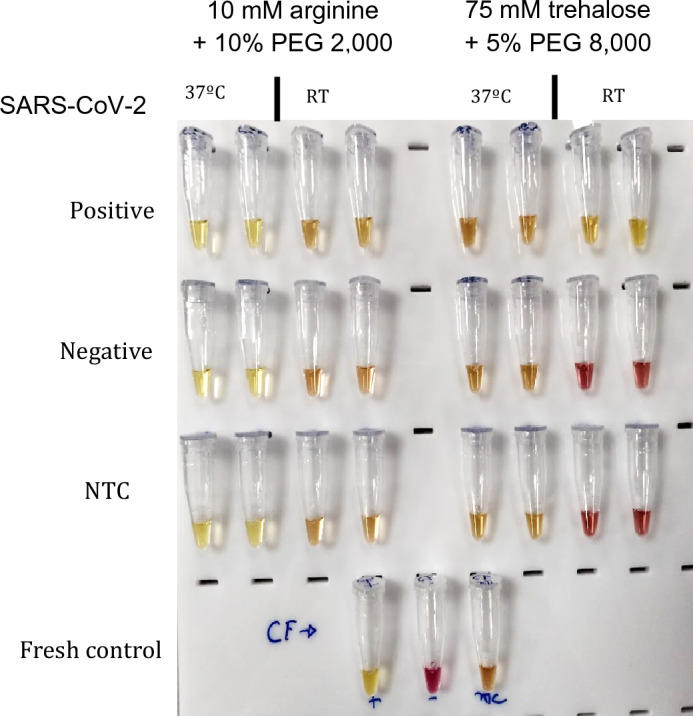
Week 2 of colorimetric SARS-CoV-2 RT-LAMP lyophilized conditions: 10% PEG 2,000 + 10 mM arginine and 5% PEG 8,000 + 75 mM trehalose. *RT* Room temperature, *NTC* Non-template control.

In subsequent tests at room temperature, the 10 mM arginine + 10% PEG 2000 condition lost its functionality by week 2, while the 75 mM trehalose + 5% PEG 8000 condition maintained its stability until week 6, marking the last observation point with sustained reaction stability (Fig. 7).

**Figure 8:**
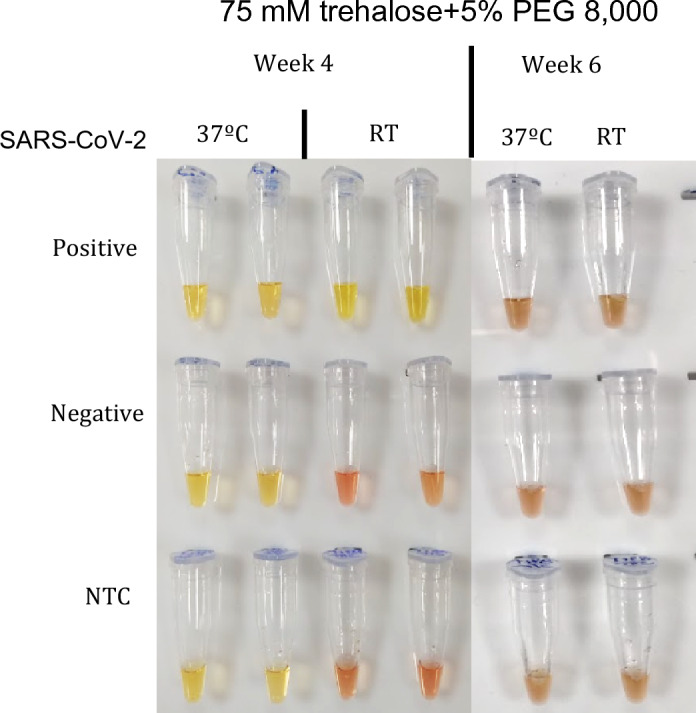
Week 4 and 6 of colorimetric SARS-CoV-2 RT-LAMP lyophilized condition with 5% PEG 8,000 + 75 mM trehalose, indicating stability at room temperature until week 4. *RT* Room temperature, *NTC* Non-template control.

The 75 mM trehalose + 5% PEG 8,000 condition was evaluated in weeks 3 (data not shown), 4, and 6, as demonstrated in Fig. 8. Week 4 reactions were the last to show stability at room temperature, successfully differentiating the color of SARS-CoV-2 positive samples from negative samples and NTC. By week 6, the same formulation stored at room temperature no longer produced clear results after 30 min in the thermocycler. Therefore, we can conclude that lyophilized colorimetric RT-LAMP with 75 mM trehalose + 5% PEG 8000 maintained stability at room temperature for at least 28 days.

## Discussion

Through a literature review focusing on classes of lyoprotectants and cryoprotective agents, selection criteria were established based on the most frequently used protective reagents in lyophilization protocols and their physicochemical properties. Trehalose is often highlighted for its ability to protect proteins through three mechanisms: hydrogen bonding between trehalose molecules and proteins for water replacement, capturing water molecules on protein surfaces to form a water layer, and preserving protein conformation within a viscous trehalose matrix for mechanical protection^8^. When compared to other additives, under certain conditions, trehalose has been defined as the best protective agent^9^. In addition to its role as a lyoprotectant and thermal protector, trehalose destabilizes duplex DNA, thereby improving the specificity and yield of isothermal amplification reactions^10^. Arginine, as an amino acid that prevents protein aggregation^11^, along with PVP and PEG, polymers recognized as effective bulking agents, were selected to ensure a homogeneous cake structure^12,13^. During testing, comparisons between concentrations and substances were made, as the selected excipients performed similar functions, which required further experimentation to determine the optimal combinations.

The preparation of a mixture of protective reagents not only combines the protective functions of each but also aims to promote a more homogeneous appearance of the freeze-dried "cake." An optimal result is evident when the cake retains the same volume and shape as the initial solution without any signs of collapse, melting, or foaming^7^. While appearance is typically more critical in large-scale freeze-drying than in laboratory applications^12^, a more homogeneous, non-scattering product may be required depending on the container used (e.g., the well of a plate).

Lyophilization of proteins is a well-established domain within scientific research, focused on enhancing structural stability and functional integrity to prolong shelf-life^12,14–17^. However, lyophilization, which aims at molecular biology and its effectiveness, is a scarce field in scientific literature. One of the earliest studies in this field involved the lyophilization of PCR Mix for the detection of *Mycobacterium bovis*, employing a standardized protocol with an optimized 5% (wt/vol) trehalose concentration^18^. In a subsequent study conducted two decades later, the optimal concentration of trehalose, as previously reported, was utilized to perform a multiplex PCR assay for the simultaneous detection of *Salmonella* spp., *Bacillus cereus*, and *Staphylococcus aureus*. This assay demonstrated no cross-reactivity with several bacterial pathogens, exhibited sensitivity similar to that of reference reports, and maintained the stability of reagents for up to two months when stored at 4 °C and for one month when stored at 25°C^19^. Another study, employing lyophilized DNA extracted from serum to detect *C. burnetii* via qPCR, showcased an enhancement in the sensitivity of qPCR within serum by concentrating bacterial DNA^20^. Despite the significant findings, these papers did not explore lyophilization protocol variables beyond trehalose concentration, such as alternative cryopreservative molecules and freezing durations. However, a recent report introduced a point-of-care PCR mix designed for room temperature use, displayed in a microfluidic format with a more complex formulation comprising trehalose, mannitol, and PEG 20,000. This formulation demonstrated stability for 1–2 years at room temperature^21^. The optimal lyophilization time emerged as a crucial variable, underscored as a pivotal component within the protocol. In our study, we observed that reactions subjected to overnight lyophilization, lasting between 17 and 18 h, experienced a loss of functionality, while reactions lyophilized for five hours also exhibited increased instability post-amplification. Additionally, Molnar et al. (2021) demonstrated lyophilization protocols for various rat and mouse tissues, indicating that lyophilized samples stored at 4 °C remained viable for up to 20 months. These samples maintained both protein and RNA quantity and quality comparable to those stored at -80 °C, thereby suggesting the invaluable utility of lyophilization in biological sample preservation^22^.

The molecular detection of SARS-CoV-2 played a crucial role in mitigating the COVID-19 pandemic, leveraging the high sensitivity and specificity of molecular diagnostic tools, particularly prior to the emergence of antigenic tests. Furthermore, the lyophilization of molecular reagents has emerged as a promising approach, as evidenced by one of the earliest reports for SARS-CoV-2 described by Xu et al. (2020). They utilized lyophilized RT-qPCR reagents containing trehalose, mannitol, BSA, and PEG 20,000, demonstrating sensitivity, specificity, and repeatability comparable to those of freshly prepared reagents. Moreover, these lyophilized reagents exhibited stability at room temperature for 28 days and at 37 °C for 14 days^13^. When lyophilization was integrated with RT-LAMP for SARS-CoV-2 detection, the initial report presented a commercial-patented formulation comprising dual-target primers utilized under an LED transilluminator. The authors found that nasopharyngeal swabs and saliva RT-PCR achieved diagnostic sensitivities of 100% and 93.33%, respectively. Nevertheless, the constraint arose from the instability of positive controls, dye, and resuspension buffer at room temperature, necessitating a cold chain but suggesting the possibility of RT-LAMP lyophilization^23^. In the same year, a study employing a commercial lyophilized RT-LAMP reagent and multiplexed primers for SARS-CoV-2 detection via turbidity measurement exhibited high diagnostic performance and no cross-reactivity with other respiratory pathogens^24,25^. However, this report does not delve into the characteristics or evaluation of the lyophilization process and is confined to the use of commercial reagents. Conversely, Colbert et al. (2022) conducted a compelling study showcasing the successful detection of SARS-CoV-2 from saliva using a lyophilized RT-LAMP approach coupled with particle imaging techniques. This detection was achieved on a portable chip with integrated heating, facilitated by a smartphone. Nevertheless, it is important to note that the primary focus of the project was not on the lyophilization process itself but rather on a fixed formulation featuring reduced betaine and a 10% concentration of trehalose^26^.

A plethora of reagents and protocols exist for developing lyophilized colorimetric RT-LAMP assays, leading to a vast array of combinations and variables. In line with this notion, Song et al. (2022) demonstrated a similar approach to our report, assessing a lyophilized colorimetric RT-LAMP method for SARS-CoV-2 detection. However, the authors only investigated trehalose and dextran in conjunction with guanidine hydrochloride, omitting arginine, PVP, or PEG from their evaluation. The lyophilization process was also standardized to one hour at 10 milliTorr and -40 ºC. Moreover, the reactions were not conducted on a thermal cycler but rather in thermoses filled with hot water at 65ºC. Despite yielding relevant analytical results, the diagnostic performance was evaluated using simulated SARS-CoV-2 infected samples, with a LoD of 15,514 viral copies per reaction. When correlated with RT-qPCR, this LoD equated to a threshold of 26.4. Subsequently, the authors analyzed the distribution of Ct values for 5,897 SARS-CoV-2 samples tested by RT-qPCR. They demonstrated that among these samples, 4,254 patients exhibited a Ct value lower than 26, resulting in a real-world sensitivity of 72%^27^. Our findings revealed a sensitivity of 100% when utilizing real SARS-CoV-2 clinical samples with Ct values below 29. Additionally, multiple lyophilized RT-LAMP reactions yielded positive results, with Ct values exceeding 29. Notably, disparities were observed in the lyophilization protocol, particularly in the freezing phase, which was conducted at − 20 °C for one hour. This freezing step is crucial in the process^28^, as alterations directly impact the procedure. Our findings revealed a sensitivity of 100% when utilizing real SARS-CoV-2 clinical samples with Ct values below 29. Additionally, multiple lyophilized RT-LAMP reactions yielded positive results, with Ct values exceeding 29. Notably, disparities were observed in the lyophilization protocol, particularly in the freezing phase, which was conducted at -20 °C for one hour. This freezing step is crucial in the process, as alterations directly impact the procedure.

We also observed a stability period of 30 days at 4 ºC and 10 days at room temperature for the lyophilized colorimetric RT-LAMP assay. However, our findings revealed a lyophilization formulation with a stability of 28 days at room temperature. This advancement represents a significant progress in the field of lyophilization and point-of-care application strategies.

Tests conducted with clinical samples have been crucial in assessing the performance of lyophilized colorimetric RT-LAMP compared to the gold standard RT-qPCR technique. As previously demonstrated by our group in other studies^24,25^, a decline in performance occurs in colorimetric RT-LAMP assays with higher RT-qPCR Ct values. An important finding from our research was that samples with higher Ct values, indicative of a lower amount of viral genetic material, exhibited more inaccurate results. Conversely, samples with lower Ct values (below 29) displayed minimal inconsistency, likely due to their higher viral RNA content. Regarding the color indicators, the optimal color for a positive RT-LAMP reaction is yellow, with acceptable shades including light yellow and golden. However, a yellowish-orange hue, bordering on positive, may introduce uncertainty during visual interpretation depending on ambient lighting conditions. For negative reactions, ideal colors include rosy red, red, and light red. Shades resembling reddish-orange may potentially result in false positive diagnoses, contingent upon lighting conditions and individual interpretation.

In the LoD tests, we also assessed the fidelity of colors post-amplification for each RNA concentration under the two selected conditions. Across all concentrations, the samples consistently maintained the same colors, transitioning from yellow to orange and red as the values decreased. Although the color discrepancy between the conditions was subtle, it was discernible. However, this discrepancy would unlikely pose an issue during clinical analysis, indicating stability in sensitivity for both conditions. Hypothetically, the combination of PEG 2,000 and arginine detected the presence of positive RNA but exhibited an orange color, potentially complicating visual analysis. Conversely, the combination with trehalose failed to detect the presence of viral RNA, suggesting it may be a less stable protective combination during lyophilization. However, while sacrificing sensitivity, combining PEG 8,000 with trehalose demonstrated greater specificity by avoiding visual interpretation uncertainties.

Additional biochemical and microfluidic options are emerging as promising tools for point-of-care tests in molecular biology^29–31^. Seok et al.^32^ described a paper-based device coupled with LAMP for real-time detection of bacterial meningitis DNA, enabling reactions in dry conditions and capable of detecting 10^2^–10^5^ copies of genomic DNA^32^. Another point-of-care paper-based test utilizing LAMP amplification combined with lateral flow nucleic acid detection strips exhibited high sensitivity and specificity for detecting hepatitis C virus in less than 40 minutes^33^. Furthermore, other reports demonstrate successful pathogen detection in paper-based devices, including *E. coli*^31^ and Zika virus^34,35^.

In conclusion, we screened and evaluated lyophilization formulations for the colorimetric RT-LAMP assay for detecting SARS-CoV-2. Our findings revealed clinical sensitivity comparable to standard RT-LAMP reactions, corroborating previous demonstrations by our group and aligning with RT-qPCR Ct values. In the lyophilization process, numerous protective agents can be employed, resulting in a multitude of possible combinations and concentrations to be assessed. Despite the limited range of protective agents and concentrations explored in this study, we identified a formulation exhibiting excellent diagnostic performance, coupled with room temperature stability lasting at least 28 days. This underscores the feasibility and real-world applicability of our methodology. Thus, this report represents a significant advancement in the lyophilization colorimetric RT-LAMP protocol, offering valuable insights for future formulations and protocols.

## Methods

### Colorimetric RT-LAMP assay

A colorimetric RT-LAMP protocol for COVID-19 detection was standardized by the Laboratory of Applied Sciences and Technologies in Health (LaCTAS) team at the Carlos Chagas Institute, which was subsequently utilized for standardizing the lyophilization process. This protocol, published in two scientific articles in 2021^24,25^, constitutes a colorimetric RT-LAMP reaction aimed at detecting SARS-CoV-2 RNA For the development of lyophilization protocols, each reaction comprises a protective agent or a combination of two agents: 12.5 μl of Master Mix WarmStart® Colorimetric LAMP (NEB, England) and 6 μl OligoMix, supplemented with 1.5 μl of Nuclease Free Water (IDT). Following lyophilization and subsequent resuspension in water, 5 μl of RNA (SARS-CoV-2 positive or negative) is added to the reaction. The master mix contains phenol red, a pH indicator that changes color from pink (negative) to yellow (positive) due to the formation of pyrophosphate ions produced during amplification, and the combination of WarmStart DNA polymerase Bst 2.0 and WarmStart reverse transcriptase RT that allow amplification of the target nucleic acid at a single temperature. OligoMix contains a set of six specific primers that target the Orf1a gene of SARS-CoV-2: FIP (Forward Inner Primer), BIP (Backward Inner Primer), FOP (Forward Outer Primer), BOP (Backward Outer Primer), FL and BL (Forward Loop and Backward Loop). For quantitative and descriptive evaluation of the method, reactions were quantified using spectrophotometry in a 384-well black plate at wavelengths of 434 nm and 560 nm (Synergy H1—BioTek®), corresponding to maximum absorption values for the reaction colors, i.e., yellow and red, at pH 6 and 8, respectively. This generated a difference between the wavelengths of yellow and red (ΔDO, i.e., 434 nm – 560 nm), with positive colorimetric RT-LAMP samples exhibiting higher ΔDO values than negative samples. Statistical analysis of ΔDO values was conducted using the t-student test, with a significance level of p < 0.05.

### SARS-COV-2 reference material

Subsequently, the extracted RNA was quantified via RT-qPCR on a LightCycler® 96 platform (Roche®, Germany) utilizing the E-gene standard curve (SARS-CoV Frankfurt1; Full virus RNA, Lot2; Institute of Virology, Charité) as a reference. This quantification process was repeated in three independent experiments, adhering to the protocol outlined by Corman et al.^36^. The RNA was then diluted to 10^7^ copies/µL, aliquoted into plastic microtubes, stored at -80ºC, thawed for use when required, and discarded after use.

### Lyophilization strategy

**Figure 9:**
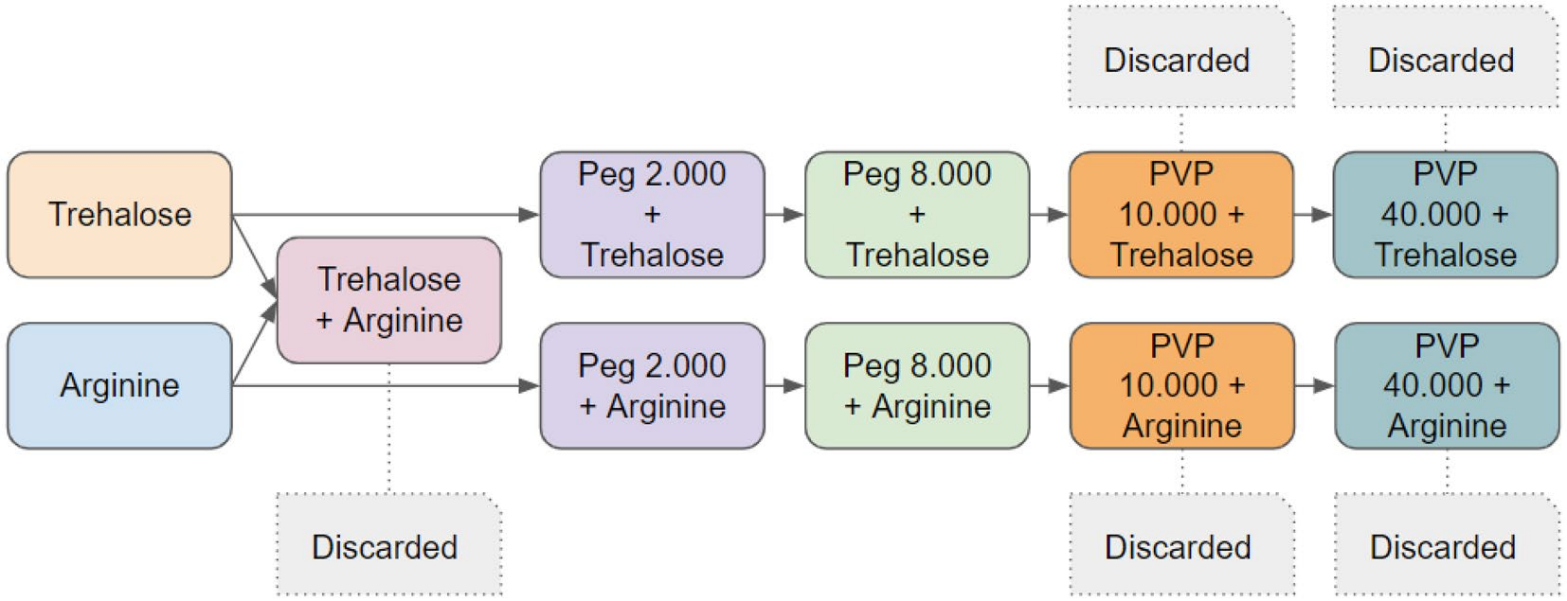
Flowchart indicating the protective reagents and combinations tested.

The colorimetric RT-LAMP conditions described in item “*Colorimetric RT-LAMP assay”* were lyophilized using the following stabilizing agents: trehalose, arginine, polyvinylpyrrolidone 40,000, PEG 2,000 and 8,000 (Sigma-Aldrich). Trehalose concentrations of 50 mM, 75 mM, 100 mM, and 150 mM were tested, while arginine concentrations of 2.5 mM, 5 mM, 10 mM, 20 mM, 30 mM, 40 mM, 72 mM, and 75 mM were chosen for testing. Concentrations of 1%, 2.5%, 8%, and 10% were tested for PEG 2,000, and concentrations of 2.5% and 5% were tested for PEG 8,000, both in association with arginine and trehalose. Two types of PVPs were subjected to testing, differing in molecular weights (10,000 and 40,000), each at concentrations of 2% and 5%, in conjunction with arginine or trehalose. Additionally, analyses were conducted to assess the effects of protective reagent concentrations, variations in procedure time (five-hour freeze-drying or overnight with a total duration of 15 h and 30 min), and combinations of protective reagents. The experimental conditions were prepared, frozen, and promptly lyophilized using a Marin Christ Alpha 2–4 LD plus equipment. The reactions were initially frozen in an ultra-freezer at − 80 ºC for 20 min before being transferred to the lyophilizer at the designated time for each test (five hours or 15 h) at a temperature of − 30ºC and a pressure of 0.37 mbar. Following lyophilization, the reactions were resuspended in 20 µL of MiliQ RNAse Free water (ThermoFisher), with the addition of 5 μL of reference RNA, SARS-CoV-2, negative control RNA, or MiliQ RNAse Free water (NTC). Subsequently, the reactions underwent thermocycling in a thermocycler (Alpha 2–4 ld plus—Martin Christ Ltda) for 30 min at a constant temperature of 65 °C. The reactions were then visually inspected, with the color shade being considered, using the naked eye. As a control in each lyophilized colorimetric RT-LAMP reaction, a "fresh control" was included, comprising the standard colorimetric RT-LAMP reaction without lyophilization. This control contained SARS-CoV-2 RNA, cell lineage RNA as a negative control and NTC (Fig. 9).

### Limit of detection

To determine an approximate Limit of Detection (LoD) with the two optimal lyophilization conditions identified, two independent assays were conducted using RNA from SARS-CoV-2 reference material quantified to 10^5^, 10^4^, 10^3^, and 10^2^ copies per reaction. A fresh control was included in each assay, serving as a reference with samples containing fresh reagents.

### COVID-19 Clinical samples and diagnostic performance evaluation

All clinical samples utilized in this study were sourced from Hospital Erasto Gaertner, Curitiba, Paraná, Brazil, following approval by the Hospital Erasto Gaertner ethics committee (CAAE 31592620.4.1001.0098). All sample collection and experimental conduction were carried out in accordance with relevant guidelines and Brazilian regulations. Informed consent was obtained from all recruited subjects and/or their legal guardians. Nasopharyngeal swabs were collected from individuals suspected of SARS-CoV-2 infection, and RNA extraction was performed using the QIAmp viral RNA mini kit (Qiagen) according to the manufacturer's instructions. SARS-CoV-2 detection was carried out via RT-qPCR using the Corman protocol^36^, which employs primers and probes targeting the SARS-CoV-2 E-gene (FAM) and the internal control human RNaseP (HEX). The reaction was conducted using a LightCycler96 instrument (Roche), with cycling parameters set at 50ºC for 30 min, 95ºC for five minutes, followed by 45 cycles of 95ºC for 15 s and 58ºC for 30 s. Samples with a Ct value below 35 for the E-gene were deemed positive, while undetectable samples that exhibited a Ct value for RNAse P below 35 were also considered positive.

Thirty positive samples for SARS-CoV-2 were selected and subdivided into two groups: 20 samples with an E-gene CT less than 29 and 10 samples with a CT between 29 and 35. Additionally, 20 samples that were undetected for the E-gene but exhibited amplification of the internal control, indicating the presence of RNA, were selected. These 50 samples underwent diagnostic evaluation by lyophilized colorimetric RT-LAMP under the two selected conditions.

### Assay stability

For the stability test, reactions were prepared and lyophilized for five hours under the selected conditions: 10 mM arginine + 10% PEG 2,000 and 75 mM trehalose + 5% PEG 8,000. A total of 132 tubes were lyophilized for testing at eight intervals: week 0, week 1, week 2, week 4, week 6, week 8, week 10, and week 12. These tubes were stored in sealed aluminum packets, each containing six freeze-dried tubes along with a 1 g silica pack. They were stored in cardboard boxes and placed in separate locations based on the temperature to which they would be exposed. Groups designated for room temperature storage were arranged in a cabinet within the laboratory, while the second group was stored in an incubator at 37 °C. The packages were opened only on scheduled testing dates, and after opening, resuspension in ultrapure water, RNA addition, and submission to the amplification protocol were performed. Stability tests utilized 5 μl of RNA from a pool of positive samples with a Ct below 28. Additionally, 5 μl of RNA from a pool of negative samples and 5 μl of nuclease-free water were included. The primary evaluation focused on product stability, with an analysis of shelf life at room temperature.

### Supplementary Information

Supplementary Figures.

## Data Availability

All data generated or analyzed during this study are included in this published article.
