# Supplementary material for: Development and evaluation of a lyophilization protocol for colorimetric RT-LAMP diagnostic assay for COVID-19
Source: Sci Rep. 2024 May 9;14:10612. doi: 10.1038/s41598-024-61163-7 (PMC11078981; doi:10.1038/s41598-024-61163-7)
Supplement: Supplementary file 1 — Supplementary Figures. [file 41598_2024_61163_MOESM1_ESM.docx]

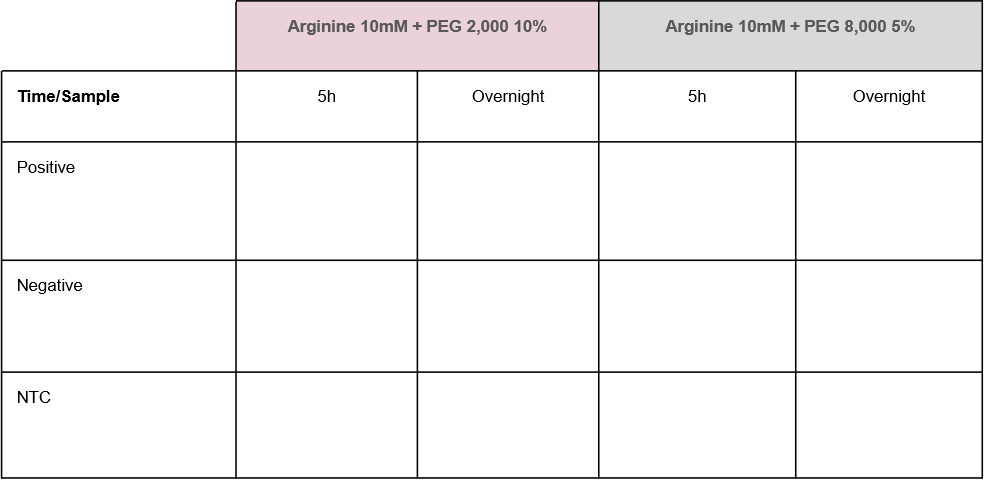

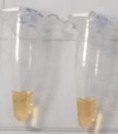

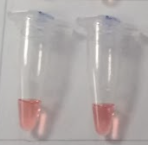

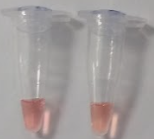

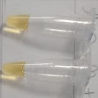

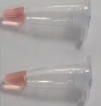

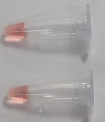

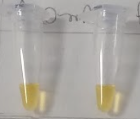

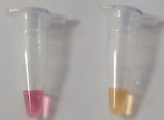

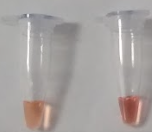

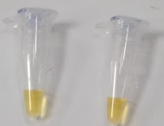

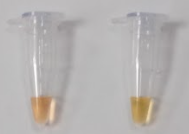

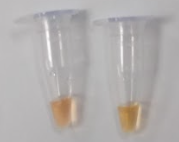


Figure S1-A: Representative figure of 5 hours and overnight lyophilization protocols of Arginine 10mM associated with PEG2,000 and PEG8,000, showing results in SARS-CoV-2 positive, negative and NTC.


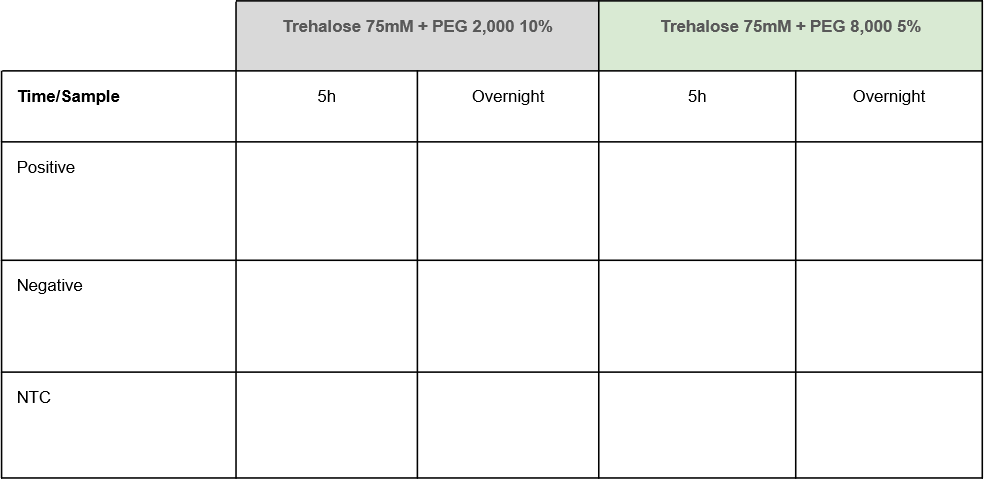

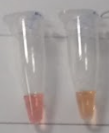

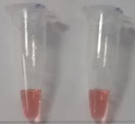

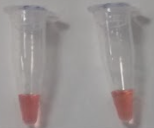

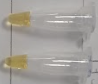

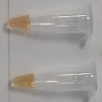

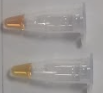

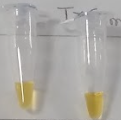

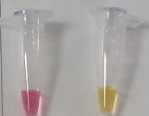

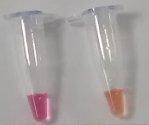

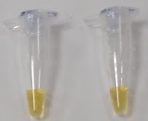

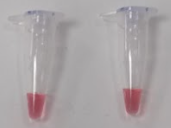

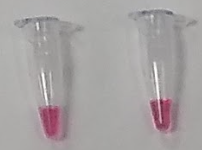


Figure S1-B: Representative figure of 5 hours and overnight lyophilization protocols of trehalose 75mM associated with PEG2,000 and PEG8,000, showing results in SARS-CoV-2 positive, negative and NTC.
